# Altered Pallidocortical Low-Beta Oscillations During Self-Initiated Movements in Parkinson Disease

**DOI:** 10.3389/fnsys.2020.00054

**Published:** 2020-07-23

**Authors:** Jeong Woo Choi, Mahsa Malekmohammadi, Hiro Sparks, Alon Kashanian, Katy A. Cross, Yvette Bordelon, Nader Pouratian

**Affiliations:** ^1^Department of Neurosurgery, University of California, Los Angeles, Los Angeles, CA, United States; ^2^Department of Neurology, University of California, Los Angeles, Los Angeles, CA, United States; ^3^Department of Bioengineering, University of California, Los Angeles, Los Angeles, CA, United States; ^4^Brain Research Institute, University of California, Los Angeles, Los Angeles, CA, United States

**Keywords:** Parkinson disease, self-initiated movements, basal ganglia thalamocortical network, local field potential, beta oscillations

## Abstract

**Background:**

Parkinson disease (PD) patients have difficulty with self-initiated (SI) movements, presumably related to basal ganglia thalamocortical (BGTC) circuit dysfunction, while showing less impairment with externally cued (EC) movements.

**Objectives:**

We investigate the role of BGTC in movement initiation and the neural underpinning of impaired SI compared to EC movements in PD using multifocal intracranial recordings and correlating signals with symptom severity.

**Methods:**

We compared time-resolved neural activities within and between globus pallidus internus (GPi) and motor cortex during between SI and EC movements recorded invasively in 13 PD patients undergoing deep brain stimulation implantation. We compared cortical (but not subcortical) dynamics with those recorded in 10 essential tremor (ET) patients, who do not have impairments in movement initiation.

**Results:**

SI movements in PD are associated with greater low-beta (13–20 Hz) power suppression during pre-movement period in GPi and motor cortex compared to EC movements in PD and compared to SI movements in ET (motor cortex only). SI movements in PD are uniquely associated with significant low-beta pallidocortical coherence suppression during movement execution that correlates with bradykinesia severity. In ET, motor cortex neural dynamics during EC movements do not significantly differ from that observed in PD and do not significantly differ between SI and EC movements.

**Conclusion:**

These findings implicate low beta BGTC oscillations in impaired SI movements in PD. These results provide a physiological basis for the strategy of using EC movements in PD, circumventing diseased neural circuits associated with SI movements and instead engaging circuits that function similarly to those without PD.

## Introduction

Patients with Parkinson disease (PD) have difficulty in initiating movements, particularly when those movements are self-initiated (SI) (Wu et al., 2011; Taniwaki et al., 2013; Jia et al., 2018). This motor impairment is related to a wider constellation of motor symptoms in PD, including rigidity and bradykinesia which result from dysfunction of the basal ganglia thalamocortical (BGTC) network (Kühn et al., 2009; Little et al., 2012; van Wijk et al., 2016). Despite these known impairments, PD patients are able to overcome deficits in movement initiation, at least in part, when an external cue is visually or aurally presented (Jahanshahi et al., 1996; Rogers et al., 1998; Martinu and Monchi, 2013; Distler et al., 2016). In order to understand the neural mechanism of this paradoxical phenomenon, several neuroimaging studies have tried to explore how brain structures within the motor network in PD are differentially involved in SI movements compared to externally cued (EC) movements (Ballanger et al., 2008; Wu et al., 2011; Taniwaki et al., 2013). A common hypothesis is that another motor circuit, namely the cortico-cerebellar network, underlies EC movements. This circuit might be relatively preserved in PD, allowing the motor system to bypass the disrupted BGTC network and lead to more normal motor performance with EC movements relative to those associated with SI movements (Ballanger et al., 2008; Wu et al., 2011; Taniwaki et al., 2013).

In general, the suppression of beta oscillations across the BGTC network prior to movement initiation (i.e., event-related desynchronization, ERD) is considered a prerequisite for motor execution (Pfurtscheller and Aranibar, 1979; Engel and Fries, 2010; van Wijk et al., 2017). However, PD patients demonstrate alterations in the beta-band ERD during movement initiation and execution which have been attributed to excessive resting beta-band synchronies (Magnani et al., 2002; Doyle et al., 2005; Crowell et al., 2012; Rowland et al., 2015; Kondylis et al., 2016). For example, Doyle et al. compared the movement-related beta ERD in the subthalamic nucleus (STN) during SI movements in both dopaminergic ON and OFF states in PD, showing that the onset of beta band ERD prior to movement onset was shortened in the unmedicated state compared to the medicated state (Doyle et al., 2005). This finding was similarly reported at sensorimotor cortical regions (Magnani et al., 2002). During EC movements, the motor cortical beta ERD has been reported to be stronger in PD compared to non-PD subjects despite similar behavioral performance (Crowell et al., 2012; Rowland et al., 2015; Kondylis et al., 2016). Bichsel et al. (2018) likewise recently reported differences in STN beta activity between EC and SI movements, where SI movements cause a generalized suppression of beta oscillations but EC movements are associated with more dynamic cue-related beta ERD. They also reported spatial segregation of movement related cortical-subcortical synchronicity between EC and SI movements, further supporting the concept that the two types of movements are mediated by functionally separated motor networks in PD patients (Bichsel et al., 2018). While there are a number of studies assessing EC and SI movements, our understanding remains incomplete due to a paucity of circuit-based analyses, clinical correlates, and non-diseased control studies, making it difficult to fully appreciate the role of distinct circuits and oscillatory signals underlying these different motor tasks.

In this study, we use invasive recordings in PD patients undergoing deep brain stimulation (DBS) implantation surgery to compare temporal changes in neural oscillatory activities within and between BG and motor cortical regions during SI and EC movements, with specific attention to how the observed differences relate to symptomatology. We compared the movement-related motor cortical dynamics with patients with essential tremor (ET), undergoing DBS implantation surgery as well, as this group is not traditionally associated with dysfunction in movement initiation. We hypothesized that SI and EC movements would be associated with distinct movement-related temporal evolution of neural synchrony profiles in BG and motor cortex and that this difference is specific to PD patients, given the specific deficits PD patients demonstrate in movement initiation. We specifically investigate the role of BG-cortical connectivity using the high temporal resolution and spatial specificity of electrocorticogram (ECoG) to understand the contribution of network synchrony to deficits in SI movements in PD. Novel to this report, we focus on the activities in globus pallidus internus (GPi) because it is the critical output node of the BGTC network.

## Materials and Methods

### Patients and Surgical Procedure

Thirteen subjects with idiopathic PD (4 females, age: 63.77 ± 7.65 years) and ten subjects with ET (6 females, age: 66.7 ± 12.56 years), undergoing DBS lead implantation surgery in the GP and the ventral-intermediate nucleus (ViM) of the thalamus, respectively, participated in this study. All subjects provided written informed consent approved by the institutional review board at the University of California, Los Angeles, in accordance with the standards set by the Declaration of Helsinki.

All PD subjects had a detailed pre-operative clinical evaluation including the Unified Parkinson Disease Rating Scale (UPDRS) motor examination (part III) performed off medication. The lateralized UPDRS-III subscores utilized for analyses were contralateral limb bradykinesia (finger tapping, hand open/close, pronation/supination, toe tapping, and leg agility) and rigidity (upper and lower extremity). All PD-related medications were withdrawn at least 12 h before the surgical procedure. The individual demographic and clinical characteristics for both cohorts are shown in Table 1. The detailed scores of sub-categories of the lateralized UPDRS-III in PD cohort are shown in Supplementary Table 1.

**TABLE 1:** Demographics and clinical characteristics in PD and ET cohorts.

**Gender/Age**		**DBS target**	**ECoG side**	**Hand mov side**	**Handed-ness**	**Preoperative total UPDRS-III**	**Preoperative lateralized UPDRS-III**		
							**Bradykinesia**	**Rigidity**	**Tremor**
**Parkinson’s disease (PD) cohort**									
1	M/72	bGP	R	L	R	31	6	4	0
2	M/58	bGP	R	L	R	72	10	3	7
3	M/60	bGP	R	L	R	35	10	4	1
4	M/69	bGP	R	L	R	52	13	5	0
5	F/61	bGP	R	L	R	32	10	4	0
6	M/61	bGP	R	L	R	37	6	3	0
7	F/73	bGP	R	L	R	9	5	1	0
8	F/57	bGP	R	L	n/a^a^	51	12	3	0
9	M/68	bGP	R	L	R	32	10	4	0
10	M/52	bGP	R	L	L	36	7	4	0
11	F/77	rGP	R	L	R	40	9	2	4
12	M/55	lGP	L	R	R	8	1	1	0
13	M/66	bSTN	R	L	R	26	1	1	6
**Essential tremor (ET) cohort**									
1	M/76	bVIM	R	L	R				
2	M/79	bVIM	R	L	R				
3	F/60	bVIM	R	L	R				
4	F/75	rVIM	R	L	R				
5	F/64	rVIM	R	L	L				
6	F/37	lVIM	L	R	R				
7	M/60	lVIM	L	R	R				
8	F/67	lVIM	L	R	R				
9	M/77	lVIM	L	R	R				
10	F/72	lVIM	L	R	R				

*DBS, deep brain stimulation; ECoG, electrocorticogram; bGP, bilateral GP; rGP, right GP; lGP, left GP; bSTN, bilateral STN; UPDRS, the unified Parkinson disease rating scale; VIM, ventral-intermediate nucleus. ^*a*^We could not identify the handedness of the subject 8 in PD cohort.*

All subjects had clinical pre and postoperative imaging. Preoperative imaging included T1-weigted MRI and a full head CT scan with the stereotactic frame. All trajectories of DBS lead implantation were confirmed with intraoperative awake macrostimulation testing, followed by a single view lateral fluoroscopy image to confirm DBS electrode placement. For bilateral cases, before right-sided DBS lead implantation, an eight-contact ECoG strip was implanted subdurally via the right frontal burr hole placed for DBS implantation. For unilateral cases, the ECoG strip was placed over the hemisphere ipsilateral to the DBS implant. Following the procedure, a postoperative CT scan was obtained for additional confirmation of the DBS electrode position.

**FIGURE 1:**
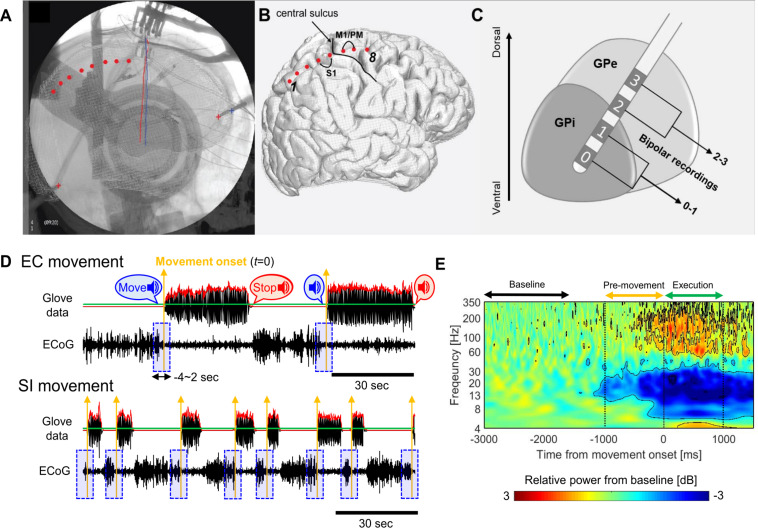
**(A)** Tips of stereotactic frame (‘+’) and DBS leads (red/blue solid lines) as landmark for the fusion of fluoroscopic image and cortical surface. Cortical contacts are marked manually on the fused images (red dots). **(B)** Marked ECoG contacts on the cortical surface relative to central sulcus and bipolar contact pairs on M1/PM. **(C)** DBS lead penetrating though GP and bipolar contact pairs in the GP. **(D)** Examples of the glove data and raw ECoG waveforms during EC (upper, 2 move/rest blocks) and SI (lower, 8 move/rest blocks) movements for one subject (red solid lines: the envelope of the averaged glove data over 5 fingers, green solid lines: threshold, 5% of the maximum value of the overall envelope, yellow upward arrows: movement onset for each block, blue shaded boxes: single-trial ECoG waveforms segmented during the –4∼2 s from movement onset for each block). **(E)** ERSP map aligned to the movement onset, which is obtained by averaging over GPi and PM/M1 contacts, movement tasks, and subjects in PD. Black contours on the map indicate the significant changes relative to the baseline activities, which exceeds the threshold values (smaller than –0.94 dB or lager than 0.77 dB).

Pre/postoperative CT scans were co-registered to preoperative structural MRI. The cortical surface was reconstructed from the MRI. A 3D surface of skull and stereotactic frame was rendered from co-registered preoperative CT scan. DBS electrodes were reconstructed from the postoperative CT scan and stereotactic frame landmarks were identified on the co-registered preoperative CT scan. The 2D intraoperative fluoroscopic image and 3D skull surface were visually inspected and fused. Reconstructed DBS leads and stereotactic frame landmarks were used to ensure maximal accuracy of 3D/2D fusion to the fluoroscopic image (Figure 1A). ECoG contacts were manually marked on the fused images and visualized on the reconstructed cortical surface (Figure 1B). The bipolar signal pair were used for the analysis of PM/M1 (premotor/motor) activities (contact pair: immediately anterior to the central sulcus, spanning precentral gyrus). The details of imaging, DBS lead targeting, and anatomical localization of ECoG strip are provided in our prior publications (Tsiokos et al., 2017; Malekmohammadi et al., 2018b, a).

### Experimental Protocol and Data Recording

We used opening/closing of the hand as testing of alternating hand movements is a typical part of examining symptoms in patients with PD. Moreover, this task was simply for implementation and tracking in the acute intraoperative setting. All subjects performed both EC and SI hand opening/closing tasks. For the EC tasks, subjects were verbally prompted to open and close their hands repeatedly with maximum amplitude at his or her fastest comfortable speed for the duration of each 30 s movement block and then verbally prompted to take a rest for 30 s until the start of the next movement block. For the SI task, subjects were instructed to initiate and terminate the same movement task independently (i.e., without external prompting) over a period of 3 min. Thus, we anticipated that the frequency of hand opening/closing for each movement block would vary across subjects but be similar between two movement conditions whereas the duration of each block and the number of movement blocks would be different (for detail, see Supplementary Figure S1). All subjects were tested with the contralateral hands to the ECoG strip side as shown in Table 1. In PD cohort, 2 and 10 subjects (of 13 subjects) were tested with dominant and non-dominant hands, respectively. In ET cohort, 6 and 4 subjects (of 10 subjects) were tested with dominant and non-dominant hands, respectively.

During the task, local field potential (LFP) from GPi and ECoG recordings were obtained using BCI2000 with g.USBamp 2 amplifiers (g.Tec, Austria, sampling rate: 2400 Hz, 0.1–1000 Hz bandpass filter, scalp ground and reference). LFPs were recorded with the DBS lead in final implant position in all subjects using the lead’s four ring electrode contacts (Medtronic 3387, Medtronic Inc., MN, contacts 0, 1, 2, and 3, ventral to dorsal, contact diameter: 1.27 mm, contact length: 1.5 mm, inter-contact distance: 1.5 mm). The cortical signals were recorded via an 8-channel ECoG strip (AdTech Medical, 4 mm contacts with 1 cm spacing) covering the motor area that was introduced subdurally in a posterior direction through the implant burr hole.

For further analyses of LFP and ECoG signals, bipolar re-referencing was used. The GPi signals (only in PD cohort) were obtained by the difference between the two deepest contacts (0 and 1) in DBS lead (Figure 1C). This was based on our previous reports that, in the GPi, the deepest contacts (at target) always have the highest spectral power in the beta and high gamma range (Tsiokos et al., 2013, 2017). The PM/M1 signals in both PD and ET cohorts were obtained by the difference between two contacts immediately anterior to central sulcus in the ECoG strip (Figure 1B). Simultaneously, hand movement was recorded using a glove with five piezoelectric sensors to measure the flexion and extension in each finger (5DT data glove 5 Ultra). The glove data were used to determine the timing of movement onset in each block as described below.

### Data Pre-processing

All signal analyses were performed using custom-written scripts in MATLAB (Version 8.6, The Mathworks Inc., Natick, MA, United States), and the open source toolboxes, Fieldtrip (Oostenveld et al., 2011) and EEGLAB (Delorme and Makeig, 2004). As shown in Figure 1D, we determined the movement onset for each block from the glove data as follows: The 5 time-series of finger movement data (corresponding to each finger) were bandpass filtered in the 1∼5 Hz (1st order zero-phase butterworth filter), and then, averaged over 5 fingers (black solid line in the upper panel in Figure 1D). The envelope of the averaged time-series was obtained by taking the absolute value of its Hilbert transform (a red solid line in Figure 1D). A threshold was determined as 5% of the maximum value of the overall envelope (a green solid line in Figure 1D), and then, a time point when the envelope is over the threshold was determined as the movement onset (t = 0) in each block of hand opening/closing (the yellow vertical arrows in Figure 1D).

A bandpass filter (1∼500 Hz, 4th order zero-phase butterworth filter) and bandstop filter (60 Hz and its harmonics, Fieldtrip toolbox) were applied to LFP and ECoG in order to reduce background noise and powerline interference. Filtered signals were then down-sampled to 1200 Hz. Single-trial LFPs and ECoGs were segmented during the −4∼2 s period relative to the movement onset for each hand opening/closing block for each movement task (the blue shaded boxes in Figure 1D). On average, 3.5 ± 0.9 EC trials and 14.8 ± 5.8 SI trials were potentially available for analysis for each PD subject. Similar numbers of trials were available in the ET cohort, including 3.5 ± 0.7 and 14.3 ± 8.9 trials, respectively. For SI movement, we discarded trials not having at least 3 s of rest prior to movement onset in order to avoid the confounding by beta rebound from prior movement termination. Although all trials were visually inspected to ensure no drift, discontinuity or the transient activity with extremely high amplitude, no trials were excluded for such artifacts. After these steps, the numbers of trials remaining per subject were 3.5 ± 0.9 and 8.6 ± 4.1 for EC and SI movements, respectively, for the PD cohort and 3.5 ± 0.7 and 6.7 ± 3.9 for the ET cohort, respectively.

### Movement-Related Oscillatory Activity Analysis

The temporal dynamics of cortical and subcortical neural oscillatory activities were investigated by an event-related spectral perturbation (ERSP) analysis. We employed Hilbert-filtering method as follows (Voytek et al., 2013): First, the single-trial waveforms were bandpass filtered in multiple and logarithmically spaced frequency bands from 0.5 to ∼400 Hz, which were partially overlapped. The successive pass bands were calculated as follows:

$$f_{L}\,\left(K\right)=0.85\,\left(f_{H}\,\left(K-1\right)\right)\,\text{and}$$

$$f_{L}\,\left(k\right)=1.1+\left(f_{H}\,\left(k-1\right)-f_{L}\,\left(k-1\right)\right)\,f_{L}\,\left(k\right)$$

where *f*_*L*_(*k*) and *f*_*H*_(*k*) are the low- and high-cutoff frequencies of the *k*^th^ pass band, and *k* = 2, …, 58, and *f*_*L*_(1) = 0.5 and *f*_*H*_(1) = 0.9. Then, a power envelope in each pass band was calculated by applying a Hilbert transform to each single-trial waveform and taking the squared values of their absolute values. From the power envelope time-series, single-trial ERSP map was obtained for each trial and then averaged across trials. Finally, the ERSP map was represented as the relative power ratio to the baseline power in the −3∼−1.5 s period prior to the movement onset, and transformed to Decibel (dB) by taking a log value.

For the significance test of movement-related power changes, we employed a non-parametric method referred to as “bootstrap random polarity inversion” (Grandchamp and Delorme, 2011). First, we randomly inverted the polarity for each time-frequency point on the trial-averaged ERSP map in dB level for each contact, movement task, and subject. This was repeatedly performed 2000 times, and then, averaged across GPi and PM/M1 contacts, movement tasks, and subjects, which enabled each time-frequency point to have a null distribution. When each time-frequency value of the original ERSP map averaged over GPi and PM/M1 contacts, movements, and subjects lied in the 2.5 or 97.5% tail of the null distribution, the value was considered significant at *p* < 0.05 (corrected, where FDR < 0.05).

From this procedure, we obtained two threshold values to determine the significance of ERD (−0.94 dB) and event-related synchronization (ERS, 0.77 dB), and these values were represented as the black contours on the ERSP map as shown in Figure 1E. Based on these significant changes, we define time and frequency ranges of interest for the further analyses. In the temporal domain, we define two distinct time periods as pre-movement (−1∼0 s) and movement execution (0∼1 sec) periods relative to the movement onset. With respect to oscillatory frequencies, we focused on two distinct frequency bands as the low-beta (13–20 Hz) and high-beta (20–35 Hz) consistent with previous literatures (van Wijk et al., 2016, 2017; Malekmohammadi et al., 2018b).

### Movement-Related Coherence Analysis

In PD cohort, movement-related coherence between GPi and PM/M1 were computed using 500 ms moving windows in steps of 250 ms (i.e., the temporal resolution is 250 ms) for each single trial. Within a window, the coherence between two time-series, *x* and *y* was calculated as follows:

$$C\,o\,h_{x,y}=\left|\left(\sum_{t=1}^{n}X\,\left(t\right)\,Y*\left(t\right)\right)\right|/\sqrt{\left(\sum_{t=1}^{n}X\,\left(t\right)\,X*\left(t\right)\right)\,\left(\sum_{t=1}^{n}Y\,\left(t\right)\,Y*\left(t\right)\right)}$$

where *X*(*t*) is an instantaneous spectrum of time-series, *x*, at a time sample, *t*, and *n* denotes the number of time samples within a window (here, *n* = 600), and the asterisk (^∗^) denotes the conjugate complex values. The instantaneous spectrum can be obtained by the Hilbert-filtering method as described above. However, the complex numbers were taken after the Hilbert transform instead of the absolute values.

A time-resolved coherence map was obtained for each single trial by calculating the coherence for overall windows and then averaging across trials. The coherence value for each time-frequency sample was calculated as the percent change relative to the baseline activities (−3∼−1.5 s period prior to the movement onset). Finally, we normalized the trial-averaged time-resolved coherence map relative to 500 surrogate data set obtained from shuffling the temporal structures of one signal of a pair of time-series, and then derived z-scores by subtracting the mean and dividing by the standard deviation of the surrogate data.

### Statistical Analyses

Non-parametric Wilcoxon singed-rank test was used to evaluate the difference between EC and SI movements (MATLAB function *signrank.m*). For the comparison between PD and ET cohorts, non-parametric Wilcoxon rank sum test was used (MATLAB function *ranksum.m*). Within the PD cohort, the correlation between neural activities and clinical characteristics was evaluated by Spearman rank correlation method (MATLAB function *corr.m*). All statistics with multiple comparisons were corrected so that the false discovery rate (FDR) would be under 0.05 (Benjamini and Hochberg, 1995).

## Results

### Comparison of Movement Kinematics

**FIGURE 2:**
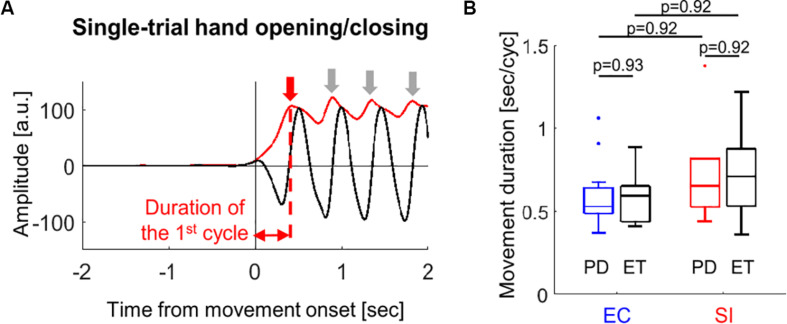
**(A)** An example of estimation of the movement duration from the single-trial glove data (block solid line) recorded during hand opening/closing for one subject (red solid line: envelope of the waveform, downward arrows: peak point for each movement cycle). **(B)** Boxplots showing the median values over PD (blue and red boxes) and ET (black boxes) cohorts of the movement duration of the first hand opening/closing average over all trials for EC and SI movements (by Wilcoxon’s test with FDR correction).

In order to compare the movement kinematics between the two movement conditions and between two cohorts, we estimated the movement duration of the hand opening/closing for each block from the glove data. Figure 2A shows an example of the temporal evolution of single-trial glove data averaged over all fingers (black solid waveform) from one subject, which contains more than 4 cycles of hand opening/closing. We detected a peak point for each cycle (represented by the red or gray downward arrow) from the envelope of this waveform (as indicated by superimposed red solid waveform). Finally, we determined the movement duration as the duration of the 1st cycle of the hand opening/closing (second per cycle) since we are interested in only the movement initiation, i.e., the first hand opening/closing for each block.

We compared the movement duration averaged across trials between PD and ET cohorts for each movement as well as between EC and SI movements for each cohort (Figure 2B). There was no significant difference in the movement duration between cohorts [*p*(corrected) = 0.93 for EC movement, *p*(corrected) = 0.92 for SI movement]. No significant difference in the movement duration was found between EC and SI movements for each cohort, either [p(corrected) = 0.92 for PD, *p*(corrected) = 0.92 for ET].

The movement kinematics during the subsequent all hand opening/closing after initiating the movement for each block were quantified as well by using 4 additional measurements, the duration of movement block, the frequency of hand opening/closing, the amplitude of hand opening/closing, and inter-hand opening/closing variability (for details, see Supplementary Figure S1A). All movement measurements during the movement block were not statistically different regardless of the movement conditions nor cohorts (Supplementary Figures S1B,C, respectively).

### Comparison of Pallidal and Cortical Oscillations Between SI and EC Movements in PD

**FIGURE 3:**
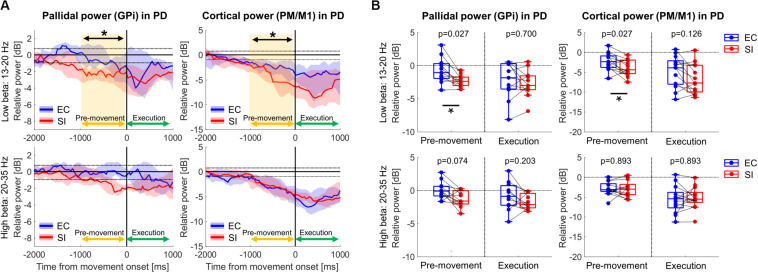
**(A)** Time-series of relative power in GPi and PM/M1 in low-beta (13–20 Hz) and high-beta (20–35 Hz) bands in PD patients (blue and red solid lines: the median values over PD patients for EC and SI movements, respectively, blue and red shades: the range from the 1st to 3rd quarter value for EC and SI movements, respectively, black dotted horizontal lines: the significance level of ERD (–0.94 dB) and ERS (0.77 dB), yellow shaded box: the significant difference between two movements). For the purpose of visualization only, a smoothing filter was applied (500 ms window). **(B)** Boxplots showing the median values over PD patients of relative power averaged within each temporal period for each contact in each frequency band (*: *p*(corrected) < 0.05 by Wilcoxon’s signed rank test, where FDR < 0.05).

Figure 3A shows the time-series of spectral power in GPi and PM/M1 in low-beta and high-beta bands in PD (*t*_0_: movement onset), which were obtained by averaging the spectral power in each frequency band on the ERSP map for each subject, and then, by taking the median value over all subjects. In both low- and high-beta bands, low and high beta ERD were observed in GPi and PM/M1 for EC and SI movements, starting prior to movement onset and lasting through movement. As shown in Figure 3B, however, a significantly larger low-beta ERD was observed for SI compared to EC movements in both GPi and PM/M1 contacts during the pre-movement period [*p*(corrected) = 0.027 for GPi, *p*(corrected) = 0.027 for PM/M1].

### Comparison of Pallidocortical Beta Coherence Between SI and EC Movements in PD

**FIGURE 4:**
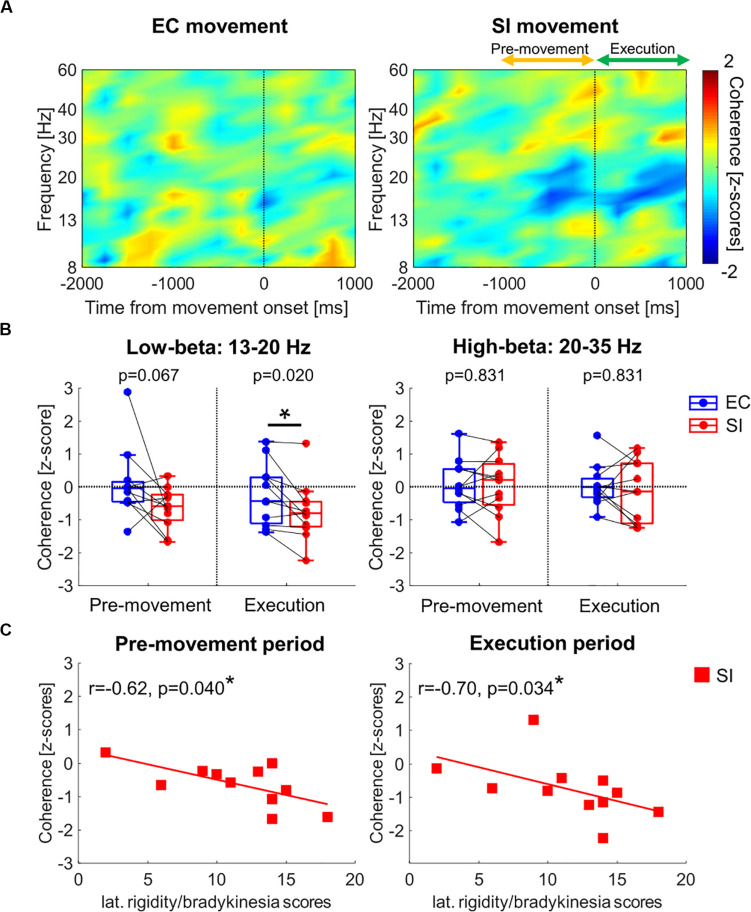
**(A)** Time-resolved pallidocortical coherence map aligned to the movement onset, which was obtained by averaging over all PD patients for each movement (frequency range: 8–60 Hz). **(B)** Boxplots showing the median values over PD patients of pallidocortical coherence averaged within each temporal period in low- and high-beta bands [*: *p*(corrected) < 0.05 by Wilcoxon’s signed rank test, where FDR < 0.05]. **(C)** Correlation analyses between clinical data (lateralized bradykinesia score) and low-beta pallidocortical coherence for SI movements in PD [*: *p*(corrected) < 0.05 by Spearman correlation, where FDR < 0.05].

Figure 4A shows the time-resolved coherence map between GPi and PM/M1 for EC and SI movements (*t*_0_: movement onset), averaged across all PD patients. Movement-related pallidocortical coherence suppression was observed mainly in the low-beta band starting ∼1 s prior to movement onset. SI movements are associated with a significantly larger reduction of low-beta pallidocortical coherence, particularly during the movement execution period (p(corrected) = 0.067 for pre-movement, p(corrected) = 0.020 for movement execution, Figure 4B). There was no significant difference in high-beta band coherence [*p*(corrected) = 0.831 for pre-movement, *p*(corrected) = 0.831 for movement execution].

### Clinical Correlations

In the PD cohort, we analyzed the correlation between pallidocortical neural activities and clinical scores in each frequency band for each movement task. Specifically, we used the lateralized UPDRS-III scores (as described in the methods section) based on the knowledge that the two body sides are not equally affected by the disease. Given that the low-beta pallidal oscillatory activities were correlated with lateralized rigidity and bradykinesia scores (Tsiokos et al., 2017), we evaluated the correlation analysis after merging the lateralized rigidity and bradykinesia score. The only significant correlation was observed between movement-related changes in low-beta pallidocortical coherence and lateralized rigidity/bradykinesia score for SI movements (as shown in Figure 4C). Specifically, subjects with more severe rigidity/bradykinesia showed stronger reduction of low-beta pallidocortical coherence during both the pre-movement and execution periods [r = −0.62, *p*(corrected) = 0.040 for pre-movement, r = −0.70, *p*(corrected) = 0.034 for movement execution]. All correlation values are shown in Table 2.

**TABLE 2:** Spearman correlation between neural activities and lateralized rigidity/bradykinesia scores in PD.

**Neural activities**	**Pre-movement**		**Movement execution**	
	**r**	**p**	**r**	**p**
**Externally cued (EC) movement**				
*Low-β power*				
GPi	–0.13	0.850	–0.71	0.060
PM/M1	–0.17	0.850	0.06	0.850
*High-β power*				
GPi	0.13	0.707	–0.22	0.707
PM/M1	0.37	0.707	0.16	0.707
Low-β coherence	–0.09	0.789	–0.26	0.789
High-β coherence	–0.03	0.936	0.09	0.936
**Self-initiated (SI) movement**				
*Low-β power*				
GPi	–0.17	0.727	–0.12	0.727
PM/M1	0.11	0.727	0.18	0.727
*High-β power*				
GPi	0.22	0.687	–0.13	0.707
PM/M1	–0.21	0.687	–0.24	0.687
Low-β coherence	−0.62*	0.040*	−0.70*	0.034*
High-β coherence	–0.29	0.381	–0.31	0.381

*Indicates statistical significance (*p* < 0.05 by Spearman correlation with multiple comparisons correction, where FDR < 0.05). GPi, Globus pallidus internus; PM/M1, premotor/primary motor cortex.

### Comparison of Cortical Oscillations Between PD and ET

**FIGURE 5:**
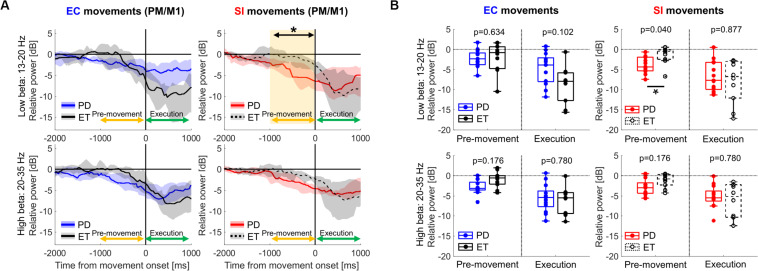
**(A)** Time-series of relative cortical power in PM/M1 in low-beta (13–20 Hz) and high-beta (20–35 Hz) for EC and SI movements (blue and red shades: the range from the 1st to 3rd quarter values of power for PD, gray shades: the range from the 1st to 3rd quarter values of power for ET, yellow shaded box: the significant difference between two cohorts). For the purpose of visualization only, a smoothing filter was applied (500 ms window). **(B)** Boxplots showing the median values of each cohort of relative cortical power averaged within each temporal period in each frequency band [*: *p*(corrected) < 0.05 by Wilcoxon’s rank sum test, where FDR < 0.05].

Finally, we assessed movement-related cortical power changes for EC and SI movements in ET patients as a control cohort (Figure 5). In contrast to that seen in PD subjects, comparison of EC and SI movements in ET patients demonstrated no difference in movement-related changes in neural oscillatory signals in PM/M1 during either pre-movement or movement execution periods (Supplementary Figure S2). Likewise, there was no significant difference in movement-related changes in neural oscillatory signals in PM/M1 between PD and ET with respect to EC movements (the left panel in Figure 5B). However, we did identify a significant difference in motor cortical low-beta ERD between PD and ET patients during pre-movement for SI movements [the right panel in Figure 5B, *p*(corrected) = 0.040].

## Discussion

SI movements in PD are associated with unique BGTC network changes during pre-movement and movement execution, both when compared to EC movements in PD and when compared to similar movements in patients with ET. Specifically, compared to EC movements, SI movements in PD demonstrate greater low-beta power suppression in GPi and motor cortex during the pre-movement period, with the motor cortical changes being unique to PD and not seen in ET subjects. SI movements in PD are also associated with greater low-beta pallidocortical coherence suppression during pre-movement and movement execution. Finally, the magnitude of low-beta pallidocortical coherence of SI movements correlates with bradykinesia severity. These unique neurophysiological observations linked to SI movements in PD are consistent with clinical observations of impaired SI in PD and are consistent with disruption of circuits related to movement initiation (Wu et al., 2011; Taniwaki et al., 2013; Bichsel et al., 2018; Jia et al., 2018).

### Altered Beta ERD in PD for SI Movements

PD subjects exhibit significant beta ERD during pre-movement and movement execution in GPi and PM/M1 regardless of movement type. This is consistent with the tenet that movement-related changes in beta oscillations within BGTC network are a prerequisite for normal motor control (Pfurtscheller and Aranibar, 1979; Engel and Fries, 2010; van Wijk et al., 2017). However, the magnitude of beta ERD in GPi and PM/M1 are significantly larger for SI movements compared to EC movements during the pre-movement period. This is similar to prior reports that ongoing habitual motor control (i.e., self-paced finger tapping) is associated with a stronger beta desynchronization in STN compared to goal-directed behavior (i.e., externally paced finger tapping) in PD patients (Bichsel et al., 2018). This has been attributed to the clinical observation that habitual motor control is more severely affected in PD (Redgrave et al., 2010). Interestingly, the significant difference in cortical beta ERD between PD and ET subjects is only evident for SI movements during the pre-movement period. Considering that in ET there is no difference in motor cortical ERDs between SI and EC movements, task-related differences in ERDs in PD can likely be attributed to the underlying pathophysiology of disease. Given that the movement kinematics did not differ between two cohorts, this may represent compensatory mechanisms to counteract excessive neural synchronies induced by the pathophysiology in PD in order to initiate the movement comparable to the subjects without PD (Rowland et al., 2015; Kondylis et al., 2016). Importantly, these differences highlight the role of BGTC circuitry in initiating movement, at least in PD if not more broadly.

### Altered Pallidocortical Beta Coherence in PD for SI Movements

Another important finding is that PD subjects demonstrate significantly larger reduction of pallidocortical beta coherence for SI compared to EC movements. In addition, this reduction of coherence for SI movements is positively correlated to the severity of motor symptom in PD (lateralized rigidity/bradykinesia score). The reduction in pallidocortical coherence in beta band is believed to be a normal neurophysiological process in movement initiation and execution (Talakoub et al., 2016; van Wijk et al., 2017). However, it has been also reported that the exaggeration in global as well as local oscillatory synchronies in beta band within BGTC network is a hallmark of PD pathophysiology (Kühn et al., 2008; Oswal et al., 2016; Malekmohammadi et al., 2018c; Wang et al., 2018). Specifically, Wang et al. reported that the pallidocortical beta coherence in resting state was relatively elevated in PD patients compared to dystonia patients, and it was suppressed by therapeutic GPi-DBS (Wang et al., 2018). Taken together, it can be interpreted that PD patients require stronger suppression of pallidocortical beta coherence in order to self-initiate movements, again highlighting the role of BGTC in initiation of movements which is exhibited both in activity-related changes in low-beta power and cross-network low-beta coherence.

In contrast to SI movements, PD patients demonstrate less reduction of pallidocortical beta coherence for EC movements and oscillatory changes related to EC movements are not significantly different than that seen in ET patients (at least in motor cortex). Moreover, ERD in PD patients for EC movements are not significantly correlated to disease symptom severity. We assume that this is because EC movements rely on circuits not reliant on the BG, such as cortico-cerebellar network, which is thought to be relatively preserved in PD patients (Glickstein and Stein, 1991; Ballanger et al., 2008; Wu et al., 2011; Martinu and Monchi, 2013; Taniwaki et al., 2013). BG plays a role to manage the movement timing, but the cerebellar circuit could take over the role of BG when the timing of the movement is signaled by external cues (Glickstein and Stein, 1991). Also, Martinu and Monchi (2013) suggested that a connection between sensory cortex and cerebellum may bypass the BGTC pathway when an external cue exists, enabling PD patients to yield almost normal motor performance. Our findings can provide further electrophysiological evidence of segregation of circuits mediating different types of movements, particularly SI vs EC movements (Bichsel et al., 2018).

### Distinct Functional Roles of Low- and High-Beta Oscillations in PD

Distinct patterns of movement-related pallidocortical oscillatory activities were observed between two sub-beta bands, low- and high-beta bands (13–20 Hz and 20–35 Hz, respectively). Specifically, the altered pallidocortical oscillatory activities for SI movements in PD were found only in the low-beta band. Given that the suppression of low-beta oscillations is thought to reflect normal motor control (van Wijk et al., 2017), this altered suppression of pallidocortical low-beta activities for SI movements can provide another robust disease-specific biomarker of the parkinsonian state. This may be particularly relevant given that the magnitude of low-beta pallidocortical coherence for SI movements is significantly correlated with the symptom severity in PD. Our findings may support the converging idea of two functionally distinct frequency ranges within the beta bands (Lopez-Azcarate et al., 2010; van Wijk et al., 2016, 2017; Tsiokos et al., 2017; Malekmohammadi et al., 2018c; Wang et al., 2018). However, the functional role of high-beta oscillations in PD remains incompletely defined and the selective modulation of low- and high-beta oscillations remains controversial (Oswal et al., 2016; van Wijk et al., 2016; Malekmohammadi et al., 2018c; Wang et al., 2018). Based on our findings, further studies exploring how the GPi-DBS modulates the altered pallidocortical oscillatory activities during SI movements would provide another important insight into the relation between the frequency-dependent alterations in BGTC oscillations and pathophysiology in PD.

### Limitations and Future Directions

Notwithstanding robust differences between SI and EC movements, there are inherent limitations to this study. First, the number of trials for each movement was small due to time limitations of intraoperative studies, potentially limiting signal-to-noise ratio which may result in type II error. Likewise, we are not able to compare results to normal physiology in subjects without disease. Instead, we employed ET patients as a control in that they do not have any dysfunction in movement initiation or motor symptom such as rigidity and bradykinesia. However, the subcortical recordings are absolutely restricted to the DBS target for each cohort, and thus, the comparison between two cohorts was only possible at the motor cortex. Considering the importance of pallidocortical networks in PD pathophysiology, comparison with dystonia patients undergoing pallidal DBS implantation would provide another important insight into the relation between the movement and pathophysiology in PD (Wang et al., 2018).

## Data Availability Statement

The raw data supporting the conclusions of this article will be made available by the authors, without undue reservation.

## Ethics Statement

The studies involving human participants were reviewed and approved by UCLA Institutional Review Board. The patients/participants provided their written informed consent to participate in this study. Written informed consent was obtained from the individual for the publication of any potentially identifiable images or data included in this article.

## Author Contributions

NP designed the research. JC, MM, AK, YB, and NP performed the experiments. NP performed the surgery. NP and YB treated patients, pre and post operation. JC, HS, and AK performed the data analysis. JC and NP wrote the first draft and all authors participated in the review and critique of the manuscript.

## Conflict of Interest

The authors declare that the research was conducted in the absence of any commercial or financial relationships that could be construed as a potential conflict of interest.
