# Supplementary material for: Altered Pallidocortical Low-Beta Oscillations During Self-Initiated Movements in Parkinson Disease
Source: Front Syst Neurosci. 2020 Jul 23;14:54. doi: 10.3389/fnsys.2020.00054 (PMC7390921; doi:10.3389/fnsys.2020.00054)
Supplement: Supplementary file 1 [file Table_1.PDF]

# *Supplementary Table*

**Supplementary Table 1**. Individual scores of sub-categories of UPDRS-III in PD patients

| | Hand move side | Preoperative lateralized UPDRS-III | | | | | | | | | | |
|---|---|---|---|---|---|---|---|---|---|---|---|---|
| | | Bradykinesia | | | | | Rigidity | | Tremor | | | |
| | | Finger tapping | Hand O/C | Hand P/S | Toe tapping | Leg agility | UE | LE | Postural (hand) | Kinetic (hand) | Rest (UE) | Rest (LE) |
| 1 | L | 1 | 1 | 1 | 2 | 1 | 2 | 2 | 0 | 0 | 0 | 0 |
| 2 | L | 2 | 2 | 2 | 3 | 1 | 1 | 2 | 2 | 2 | 2 | 1 |
| 3 | L | 3 | 3 | 3 | 1 | 0 | 3 | 1 | 0 | 0 | 1 | 0 |
| 4 | L | 3 | 2 | 3 | 3 | 2 | 2 | 3 | 0 | 0 | 0 | 0 |
| 5 | L | 2 | 2 | 2 | 2 | 2 | 2 | 2 | 0 | 0 | 0 | 0 |
| 6 | L | 1 | 1 | 2 | 2 | 0 | 2 | 1 | 0 | 0 | 0 | 0 |
| 7 | L | 2 | 1 | 1 | 1 | 0 | 1 | 0 | 0 | 0 | 0 | 0 |
| 8 | L | 2 | 2 | 2 | 3 | 3 | 1 | 2 | 0 | 0 | 0 | 0 |
| 9 | L | 2 | 2 | 2 | 2 | 2 | 2 | 2 | 0 | 0 | 0 | 0 |
| 10 | L | 2 | 2 | 1 | 1 | 1 | 2 | 2 | 0 | 0 | 0 | 0 |
| 11 | L | 2 | 2 | 2 | 2 | 1 | 2 | 0 | 1 | 1 | 2 | 0 |
| 12 | R | 0 | 0 | 0 | 1 | 0 | 1 | 0 | 0 | 0 | 0 | 0 |

| | | | | | | | | | | | | |
|---|---|---|---|---|---|---|---|---|---|---|---|---|
| 13 | L | 1 | 0 | 0 | 0 | 0 | 1 | 0 | 1 | 2 | 2 | 1 |

Abbreviation: UPDRS, the unified Parkinson disease rating scale; O/C, open/close; P/S, pronation/supination; UE, upper extremity; LE, lower extremity
