# Supplementary material for: Altered Pallidocortical Low-Beta Oscillations During Self-Initiated Movements in Parkinson Disease
Source: Front Syst Neurosci. 2020 Jul 23;14:54. doi: 10.3389/fnsys.2020.00054 (PMC7390921; doi:10.3389/fnsys.2020.00054)
Supplement: Supplementary file 2 [file Image_1.PDF]

Supplementary Figures

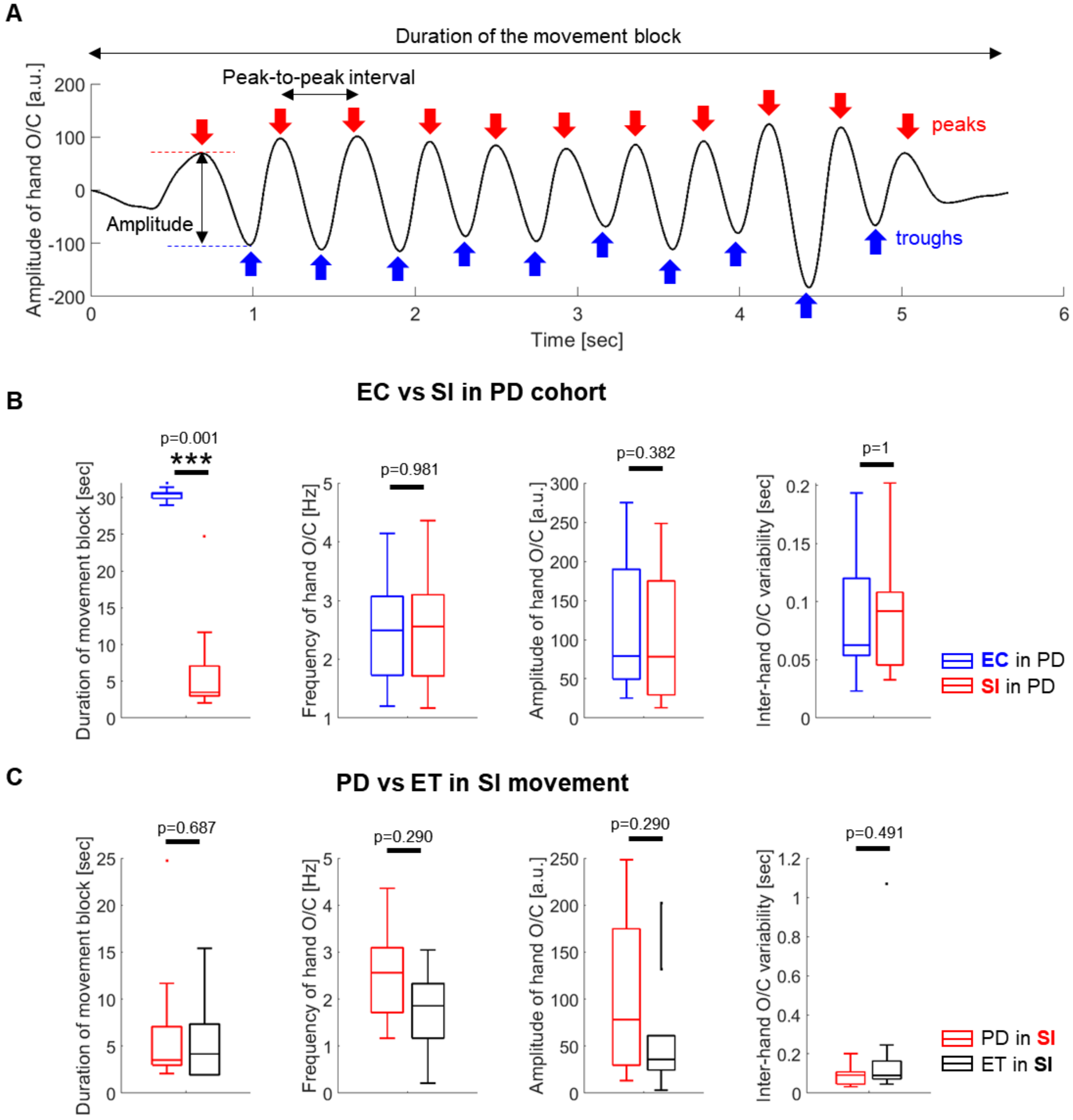

Supplement Figure 1. Movement kinematics during hand opening/closing. (A) Definition of the measures of movement kinematics from an example of glove data for one SI movement block for one subject. (B) and (C) show the statistical comparisons in the movement measures between EC and SI movement conditions and between PD and ET cohorts, respectively (***: $p<0.005$ by Wilcoxon's test where FDR<0.05).

Comparison of movement kinematics during the movement block

Supplement Figure 1A shows an example of glove data for one SI movement block for one subject and several features to define the movement measurements. The duration of movement block can be defined as the duration of all hand O/C for each movement block (5.66 seconds). The frequency of hand O/C can be defined as the number of peaks per the duration of movement block (11 peaks per 5.66 seconds = 1.90 Hz). The amplitude of hand O/C can be defined as the averaged peak-to-trough amplitudes over all hand O/C for each block (182.08). Lastly, the inter-hand O/C variability can be defined as the standard deviation of peak-to-peak intervals over all hand O/C for each block (0.03 sec).

Then, we statically compared these movement measurements between EC and SI movement conditions as well as between PD and ET cohorts (as shown in Supplement Figure 1B and C, respectively). All measurements were obtained for each movement block and then averaged over all blocks for each movement condition for each subject. We found the significantly longer duration of movement block for EC compared to SI (as shown in Supplement Figure 1B). This is not surprising because EC had a cue to initiate/terminate each block for 30 seconds but SI did not. In addition, we could not find any other significant differences in the measurements between EC and SI movements (Supplement Figure 1B) and between PD and ET cohorts in SI movement, either (Supplement Figure 1C). Overall, the actual motor performances of hand opening/closing during the movement block were similar regardless of the movement conditions and cohorts.

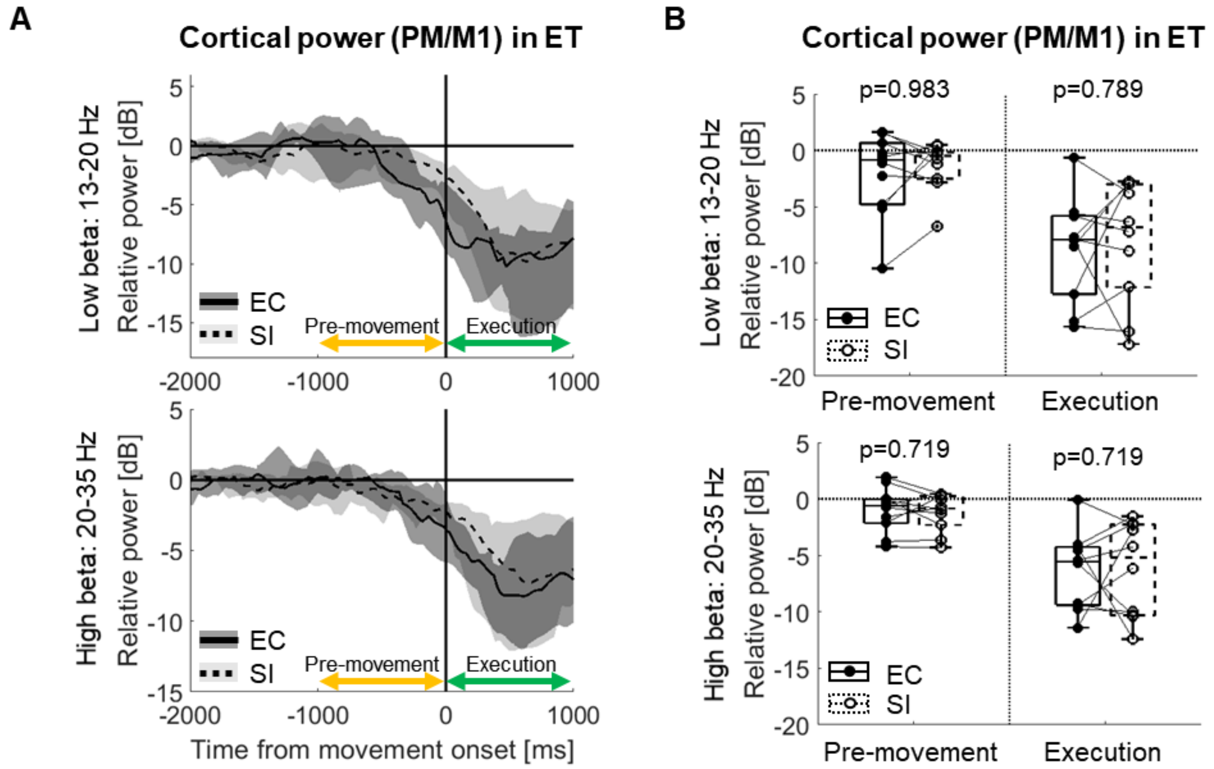

Supplementary Figure 2 (A) Time-series of relative power (in dB) in PM/M1 in low-beta (13-20 Hz, top) and high-beta (20-35 Hz, bottom) bands in ET patients. For each panel, the blue and red solid lines indicate the median values over ET patients for EC and SI movement, respectively. For the purpose of visualization only, a smoothing filter was applied (500 ms window). The darker and brighter gray shades denote the range from the 1st to 3rd quarter value for EC and SI movement, respectively. **(B)** Boxplots showing the median values over ET patients of relative power averaged within each temporal period (left: pre-movement, right: execution period) for each contact in each frequency band (by Wilcoxon's signed rank test, where FDR<0.05).
